# Single-Center Experience in the Endovascular Management of the Combination of Isolated Common and Internal Iliac Artery Aneurysms

**DOI:** 10.3389/fsurg.2021.693233

**Published:** 2021-07-15

**Authors:** Wei Wang, Jianqiang Wu, Jiang Shao, Fang Xu, Yuexin Chen, Bao Liu, Yuehong Zheng

**Affiliations:** Department of Vascular Surgery, State Key Laboratory of Complex Severe and Rare Disease, Peking Union Medical College Hospital, Chinese Academy of Medical Sciences and Peking Union Medical College, Beijing, China

**Keywords:** common iliac artery aneurysms, internal iliac artery aneurysms, endovascular procedures, stent graft, embolization

## Abstract

**Objective:** The combination of isolated common and internal iliac artery aneurysms (CIIAA) are rare, life-threatening, abnormal conditions with relatively complex treatment. This study aimed to evaluate the clinical characteristics and treatment outcomes of CIIAA.

**Methods:** We retrospectively reviewed 26 patients with CIIAA consecutively treated between January 2010 and August 2020 at Peking Union Medical College Hospital. Demographic, clinical characteristic, treatment strategy and outcome data were collected and analyzed.

**Results:** Twenty-six patients (24 men and 2 women) with a mean age of 70 years were included. There was a total of 72 aneurysms, and the mean diameters of the common iliac artery aneurysms (CIAA) and internal iliac artery aneurysms (IIAA) were 36 and 38 mm, respectively. Ten patients (38%) presented with bilateral CIAA and unilateral IIAA, and eight (31%) had CIAA with ipsilateral IIAA. All patients were treated with endovascular repair, and the overall primary technical success rate was 100%. The surgical techniques mainly included combined bifurcated stent grafting and embolization (*n* = 11), combined straight stent grafting and embolization (*n* = 8), and internal iliac artery (IIA) reconstruction (*n* = 7). There were no patient deaths or reintervention during hospitalization. The mean follow-up time was 43 months, and the patency rate of the stent was 96.2%. The overall reintervention rate was 7.7%. During the follow-up period, there were no aneurysm-related deaths.

**Conclusions:** Endovascular repair involving stent graft placement, coil embolization and IIA revascularization is a safe and effective treatment for isolated CIIAA.

## Introduction

Isolated iliac artery aneurysms (IAA) are rare clinical conditions, accounting for 0.4–1.9% of intra-abdominal aneurysms (estimated prevalence 0.008–0.03%) (1). The common iliac artery (CIA) is most frequently involved (70–90%), followed by the internal iliac artery (IIA) (10–30%), and the external iliac is infrequently involved (2). The combination of isolated common and internal iliac artery aneurysms (CIIAA) are relatively complex condition that has not been reported separately from other IAA to date. IAA are frequently asymptomatic and usually discovered incidentally, and symptomatic aneurysms are often related to the compression of adjacent structures (3, 4).

Before endovascular treatment options became available, open surgery was the standard treatment for many years (5). Due to technical advances in endovascular repair, more IAA have been repaired with endovascular intervention, and some studies on isolated common iliac artery aneurysms (CIAA) or isolated internal iliac artery aneurysms (IIAA) have suggested that elective aneurysm treatment is safe and effective in terms of morbidity and mortality (6–8).

The existing knowledge on the clinical features and treatment choices for CIIAA have mainly originated from studies on IAA. The aim of this study was to summarize the clinical features, treatment strategies and outcomes of CIIAA in our institution.

## Materials and Methods

### Patients

This retrospective study was approved by the local ethics committee, and the requirement for informed consent was waived. Twenty-six patients who were diagnosed with CIIAA treated with endovascular repair at Peking Union Medical Hospital from January 2010 to August 2020 were included.

The inclusion criteria were patients with CIIAA who were identified by searching medical record systems and for whom the diagnoses of CIIAA were established on the base of their medical history and imaging results of contrast-enhanced computed tomography or/and digital subtraction angiography scans. The exclusion criteria included dissecting CIIAA, false CIIAA secondary to inflammation, infection or trauma, CIIAA with aorta aneurysms, emergency operations and ruptured iliac aneurysms.

Each patient's medical record was reviewed, and the records included their demographic data, past medical history, clinical presentation, the results of auxiliary examinations, imaging data, iliac aneurysm characteristics, surgical details, preoperative and 30-day results, and long-term outcomes.

### Treatment Strategy

The indications for repair were the presence of at least one IAA > 30 mm in diameter, rapid enlargement of the IAA (by more than 5 mm in diameter within 6 months or 10 mm in diameter within 1 year) or symptomatic aneurysms.

The type of treatment was selected on the base of the patient's fitness and age, evaluation of clinical outcomes and effects of pelvic ischemia on quality of life, anatomic conditions for proximal and distal landing zones of the aneurysms and the anatomic suitability of IIA revascularization techniques. The endovascular technique mainly involves stent graft placement, embolization or revascularization of the IIA, which involves IIA stent graft implantation, the sandwich technique and iliac branch device (IBD), and IIA is preserved whenever possible. There was no commercially available IBD in China during the study period. The IBD we adopted in this study was surgeon-modified iliac branched device as described previously in our center (9).

We considered performing a stent graft when the CIAA or IIAA had a sufficient proximal and distal landing zone of at least 15 mm. If the CIAA with an insufficient proximal landing zone, bifurcated stent graft at aortic bifurcation would be performed. And we did not treat the IAA with a diameter of <30 mm for the purpose of preserving the IIA.

### Follow-Up

A follow-up was usually performed with computed tomography angiography or Duplex ultrasound at 1, 6, and 12 months and yearly thereafter. Clinical symptoms, potential endoleak and the patency of the stent grafts were noted at the follow-ups.

### End Points

The early endpoints were primary technical success, postoperative complications, reintervention rate and in-hospital mortality. The late end points were the patency of the stent, stent graft occlusion, the reintervention rate, long-term complications, and long-term mortality.

Primary technical success was defined as the correct deployment of the graft, with patency of the target vessels in the absence of endoleak at the end of the operation. The complications mainly included endoleak, buttock claudication and other issues related to the operation. Patency was defined as the absence of thrombosis, as assessed using either computed tomography angiography or ultrasound.

### Statistical Analysis

The categorical variables are presented as numbers and percentages, and the continuous variables are shown as means ± standard deviations. Statistical analysis was performed using SPSS, version 23 (IBM Corp, Armonk, NY).

## Results

### Patient Characteristics

A total of 26 patients (24 males, 92.3%) were included in the study, with a mean age of 70 years (range from 53 to 81 years). The primary comorbidities included hypertension (69.2%) and diabetes mellitus (26.9%). The disease course was ~3 months, and 18 patients (69.2%) were asymptomatic and were identified incidentally by ultrasound or computed tomography. The symptomatic patients presented with abdominal pain (5, 19.2%) or an abdominal mass (2, 7.7%). The patient demographics and comorbidities are listed in Table 1 in detail.

**Table 1:** Clinical characteristics of patients.

**Characteristics**	**No. or mean ± SD**	**% or range**
**Demographic characteristics**
Male	24	92.3
Age (years)	70 ± 8	(53, 81)
BMI (body mass index)	26 ± 4	(16, 33)
**Comorbidities**
Hypertension	18	69.2
Diabetes mellitus	7	26.9
Coronary artery disease	4	15.4
Peripheral artery disease	6	23.1
Benign prostatic hyperplasia	4	15.4
Previous cerebral infarction	4	15.4
Current smoking	9	34.6
Disease course (month)	3 ± 5	(1, 24)
**Symptoms**
Asymptomatic	18	69.2
Abdominal pain	5	19.2
Abdominal mass	2	7.7

### Imaging Characteristics

**Figure 1:**
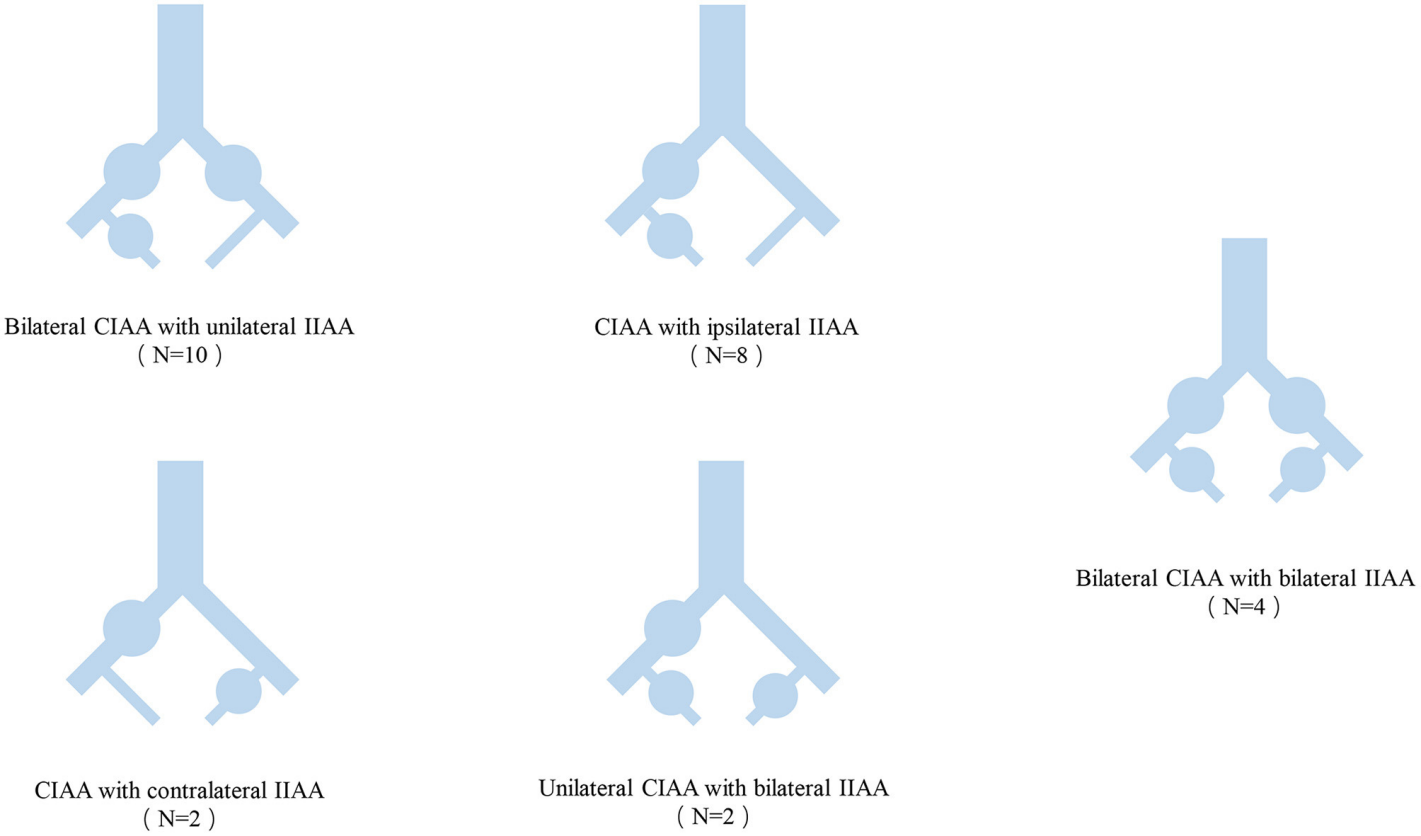
Anatomic classification of CIAA and IIAA. CIAA, Common iliac artery aneurysms; IIAA, Internal iliac artery aneurysms.

The anatomical characteristics of the aneurysms are shown in Table 2 and Figure 1. Among the 26 CIIAA that underwent repairs, there were 72 aneurysms, 40 (56%) of which were CIAA with an average diameter of 36 mm (range, 20–63 mm) and 32 (44%) of which were IIAA with an average diameter of 38 mm (range, 20–80 mm). Bilateral CIAA with unilateral IIAA were present in 10 patients (38%), and CIAA with ipsilateral IIAA were seen in 8 patients (31%). Bilateral CIAA with bilateral IIAA were seen in 4 patients (15%), and CIAA with contralateral IIAA were present in 2 patients (8%). Two patients (8%) had unilateral CIAA with bilateral IIAA.

**Table 2:** Aneurysm characteristics of the 26 patients.

**Characteristics**	**No. or mean ± SD**	**% or range**
Number of aneurysms	72	100
CIAA	40	56
IIAA	32	44
**Aneurysm diameter (mm)**
CIAA	36 ± 13	(20, 63)
IIAA	38 ± 16	(20, 80)
Intraluminal thrombus	14	53.8
**Location**
Bilateral CIAA with unilateral IIAA	10	38
CIAA with ipsilateral IIAA	8	31
Bilateral CIAA with bilateral IIAA	4	15
CIAA with contralateral IIAA	2	8
Unilateral CIAA with bilateral IIAA	2	8

*CIAA, common iliac artery aneurysms; IIAA, internal iliac artery aneurysms*.

### Management

**Figure 2:**
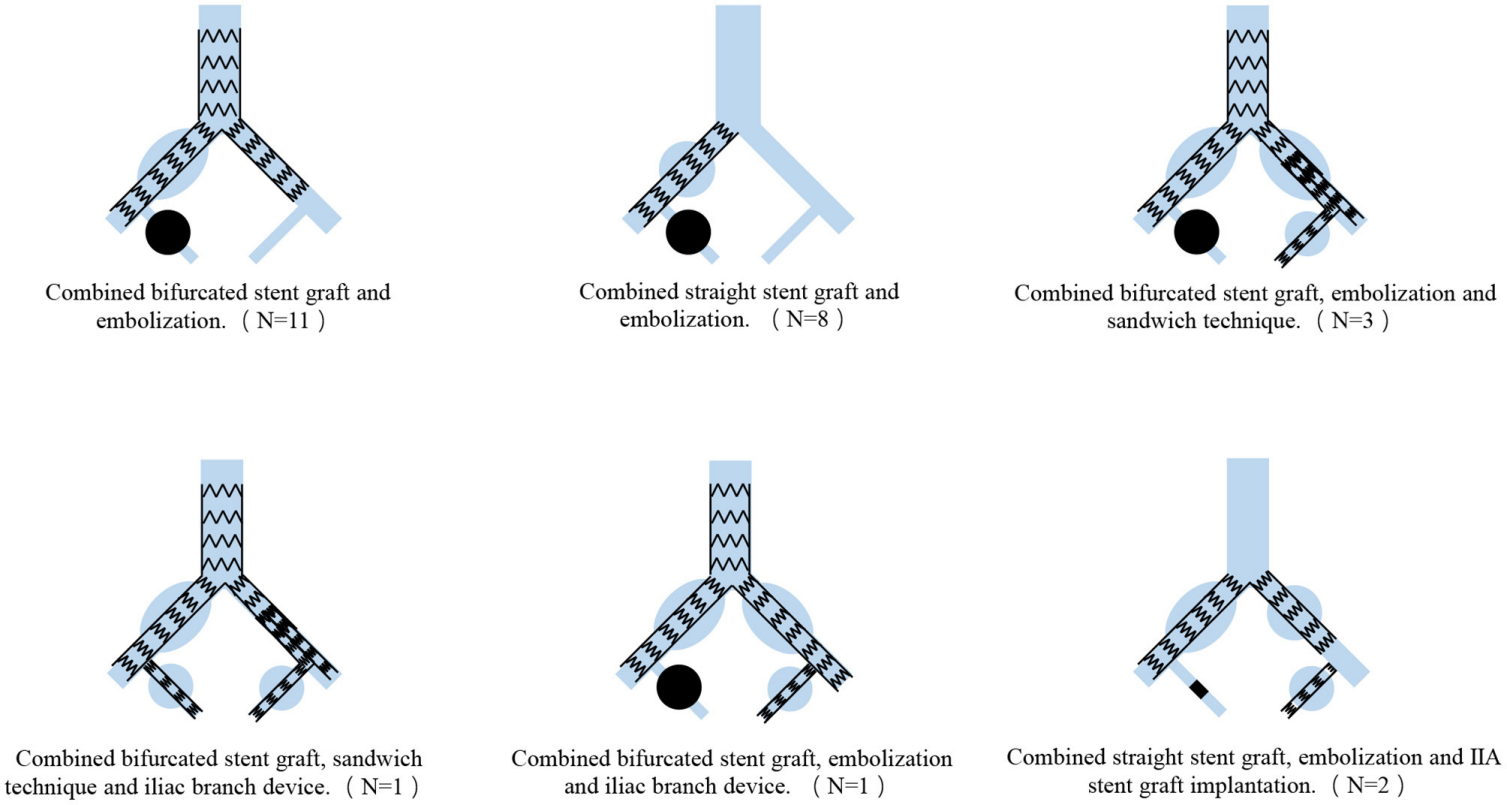
Surgery classification of the combination of isolated common and internal iliac artery aneurysms.

Sixty-six aneurysms in 26 patients were treated with endovascular repair (Figure 2). Eleven of the 26 patients were treated with combined bifurcated stent grafting and embolization, and the contralateral IIA perfusion was preserved. We treated the 8 cases with combined straight stent grafting and embolization. Three patients underwent the combination of bifurcated stent grafting, embolization and the sandwich technique to preserve the contralateral IIA perfusion. In one case with unilateral CIAA and bilateral IIAA, we used the combination of bifurcated stent grafting, the sandwich technique and IBD to preserve perfusion in the bilateral IIA. One patient had bilateral CIAA and bilateral IIAA and was treated with the combination of bifurcated stent grafting, embolization and IBD. Another 2 patients had bilateral CIAA with unilateral IIAA, and we performed repair with the combination of straight stent grafting, embolization and IIA stent graft implantation. Among all aneurysms, there were 6 aneurysms with diameters of <30 mm in 5 patients who had contralateral IIA or CIA expansion, and we did not treat these aneurysms to preserve the IIA on at least one side.

### Perioperative Results

Twenty-two patients (85%) who underwent repair were treated with general anesthesia, and the other 4 patients (15%) were treated with local anesthesia. And arteriotomy was performed in six cases due to arterial stenosis in puncture site. The technical success rate was 100%. The major in-hospital complications consisted of buttock claudication (*n* = 4), postoperative pneumonia (*n* = 1), urinary infection (*n* = 1) and postoperative fever (*n* = 1). Type Ib endoleak was found in 1 patient who underwent repair with IBD at the 30-day follow-up. The mean length of hospital stay was 13 days. None of the patients required a reintervention or died during hospitalization or the 30-day follow-up period (Table 3).

**Table 3:** Perioperative and 30-day results in 26 cases.

**Patients (*n* = 26)**	**No. or mean ± SD**
**Anesthesia**
Local	4
General	22
**Access**
Percutaneous	20
Arteriotomy	6
Primary technical success	26
**Complications**
Buttock claudication	4
Type Ib endoleak	1
Postoperative pneumonia	1
Urinary infection	1
Postoperative fever	1
Reinterventions	0
In-hospital mortality	0
Hospital stay (day)	13 ± 5

### Follow-Up

The mean follow-up duration was 43 months. During the follow-up period, the primary patency rate was 96.2%, with stent graft occlusion of the external iliac artery position of sandwich device occurring in one patient; this patient was treated with combined bifurcated stent graft, embolization and sandwich technique. Two patients underwent reintervention, one of which was due to type Ib endoleak in the IIAA after IBD implantation, and we performed balloon dilation for the second intervention. The other patient presented type Ia endoleak in CIAA after stent implantation and IIAA recurrence with blood flow originating from the contralateral IIA in the seventh year; the patient had previously undergone the combination of straight stent grafting, embolization and IIA stent graft implantation; the second operation involved bifurcated aortoiliac graft placement and coil embolization of the inflow artery. Four patients had buttock claudication; one case was treated with combined bifurcated stent graft and embolization, another one was treated with combined straight stent graft and embolization and the other two cases were treated with combined stent graft, embolization and IIA revascularization. One patient who was treated with the combined bifurcated stent graft and embolization presented with Type II endoleak in the first postoperative year. The aneurysm-related mortality rate was zero (Table 4). During the period of follow up, the six aneurysms (<30 mm) that we didn't deal with to preserve the IIA didn't appear further expansion.

**Table 4:** Long-term follow-up in 26 cases.

	**BSGE**	**SSGE**	**SGER**	**Total**
No. of treated patients	11	8	7	26
Mean follow-up period (month)	36 ± 22	48 ± 34	51 ± 22	43 ± 26
Stent graft occlusion (No.)	0	0	1	1
Reintervention (No.)	0	0	2	2
**Long-term complications**
Buttock claudication (No.)	1	1	2	4
Type Ia endoleak (No.)	0	0	1	1
Type II endoleak (No.)	1	0	0	1
Aneurysm-related mortality	0	0	0	0

*BSGE, Combined bifurcated stent graft and embolization; SSGE, Combined straight stent graft and embolization; SGER, Combined stent graft, embolization and internal iliac artery revascularization*.

## Discussion

CIIAA are rare conditions with relatively complex treatment strategies. To date, no studies have reported the clinical characteristics and treatment methods of CIIAA specifically, and this study is the largest single-center series of CIIAA on the clinical features, treatment strategies and outcomes. The article provides evidence that endovascular repair involving stent-graft implantation, embolization and IIA reconstruction is a safe and effective method of treatment for CIIAA.

Similar to CIAA or IIAA, CIIAA occur more frequently in elderly men with a history of hypertension and diabetes mellitus, and most cases are found incidentally in asymptomatic conditions during an examination (10, 11). The diameters of the CIIAA were also consistent with those reported in previous studies about CIAA or IIAA (8, 12). We found most CIIAA included bilateral CIAA with unilateral IIAA and CIAA with ipsilateral IIAA.

Clear treatment guidelines for IAA have not been established, and open surgical repair of IAA was the treatment of choice for many years until endovascular repair gained widespread acceptance (4). The endovascular technique has been reported to be safer and more effective in the short- and long-term periods and has many potential advantages (1, 5, 13, 14). In those cases, the mean length of stay is 13 days and the postoperative length of hospital stay was 5 days. A long hospital stay mainly contributed to preoperative evaluation of other chronic diseases before general anesthesia. In our study, the critical endovascular technique used to manage the CIIAA was the combination of stent graft implantation, embolization, and IIA reconstruction, and the key factors in selecting the appropriate treatment methods included the extent of the proximal landing zone in CIAA and the distal zone in CIAA and IIAA.

The treatment strategy for the proximal zone of CIAA is selected mainly on the base of the extent of the proximal landing zone; in the presence of a sufficient landing zone, straight stent grafting is a reliable method. For patients with unfavorable proximal landing zones, standard bifurcated aortic stent graft placement with limb extension may be necessary (15, 16). In this study, 16 patients received bifurcated aortic stent grafts due to inadequate proximal landing zones, and the other patients who underwent repair with endovascular methods received straight stent grafts in the proximal landing zone.

The distal zone of CIAA needs to be managed with consideration of the situation of the IIAA and IIA blood flow should be preserved at least unilaterally. If feasible, both bilateral IIAs should be preserved to prevent pelvic ischemia. Unilateral IIA embolization after careful selection may be a feasible choice under some circumstances and has the advantages of being safe, low-cost and simple in terms of the technology required. For patients with an advanced age, low activity level, high perioperative risk, ischemic symptoms that do not impact quality of life considerably, or have cases anatomically unsuitable for treatment by IIA revascularization, unilateral IIA embolization is attractive, but younger patients with a longer life expectancy and higher quality of life may substantially benefit from bilateral or unilateral IIA preservation to prevent ischemic complications (11, 17). In our study, majority of patients with CIIAA were elderly and had multiple medical complications. Most patients underwent repair with a combination of stent graft placement and IIA embolization, and for the purpose of preserving IIA flow, seven patients were treated with revascularization of the IIA.

Various endovascular techniques of IIA reconstruction, including but not limited to stent graft placement in IIA alone, the sandwich technique, and IBD, have been developed to preserve antegrade flow to the IIA and minimize the risk of pelvic ischemia (10, 18, 19). Stent graft placement into the IIA alone from the proximal to distal IIA is a potential treatment for patients with sufficient proximal and distal landing zones with a length of at least 15 mm length and diameter of at least 5 mm (11). It has been indicated that the sandwich technique and IBD are safe and effective choices for IIA revascularization; both treatments have high technical success, and no significant differences have been observed in long-term results (20). The selection between the two techniques mainly depends on the anatomical features and surgeon's experience. In our series, four patients were treated with the sandwich technique. IBD was performed in 2 patients, and 2 patents underwent stent graft placement into the IIA alone.

Endoleak was identified in 3 patients after endovascular treatment. Among them, 1 patient developed a Type 1b endoleak in the distal end of the IIA after IBD was implanted, and the patient underwent reintervention with stent balloon dilatation. Type II endoleak occurred in 1 patient after bifurcated aortoiliac graft placement with IIA embolization. This case of endoleak was caused by retrograde flow from the lumbar arteries and did not result in the development of an aneurysm due to low flow during the follow-up period. Another patient presented with type 1a endoleak after stent-graft placement with IIA embolization due to the formation of an abdominal aorta aneurysm and its extension to the ICA in the seventh-year follow-up, and reintervention with bifurcated aortoiliac graft placement was performed.

Buttock claudication was the most common complication that occurred after IIA embolization (21). In our series, 4 patients (15.4%) experienced buttock claudication; two patients were repaired with the combination of stent graft placement and IIA embolization; one patient was treated with combined bifurcated stent graft, embolization and sandwich technique; another patient was repaired using combined straight stent graft, embolization and IIA stent graft implantation. And all of them suffered from buttock claudication at the side of IIA embolization; Overall, the incidence rate of buttock claudication was relatively low compared to previous reports (8). This discrepancy may be attributed to the preservation of at least one path of flow to the IIA and embolization being performed as proximal as possible from the aneurysm sac origin.

This study has several limitations. This was a retrospective, non-randomized study that only included a single center, and the sample size was small. In addition, the selection of the therapeutic schedule was based on the clinical judgment of each treating physician. Thus, additional larger and preferably randomized clinical studies with longer follow-up periods are needed to evaluate the effects of endovascular repair.

## Conclusion

Endovascular treatment of CIIAA with the combination of stent-graft placement, embolization and IIA revascularization is safe and effective, with a high technical success rate and low mortality. The treatment strategy should be mainly based on the anatomical features of the CIIAA, especially the proximal landing zone of the CIAA and management of the IIA.

## Data Availability Statement

The raw data supporting the conclusions of this article will be made available by the authors, without undue reservation.

## Ethics Statement

The studies involving human participants were reviewed and approved by Ethics Committee of Peking Union Medical College Hospital (JS-2629). The patients/participants provided their written informed consent to participate in this study. Written informed consent was obtained from the individual(s) for the publication of any potentially identifiable images or data included in this article.

## Author Contributions

WW, JS, and YZ: conception and design, analysis, and interpretation. WW, JW, and FX: data collection and statistical analysis. WW, JS, and JW: writing the article. WW, YZ, JS, YC, and BL: critical revision of the article. YZ: overall responsibility. All authors read and approved the final version of the manuscript.

## Conflict of Interest

The authors declare that the research was conducted in the absence of any commercial or financial relationships that could be construed as a potential conflict of interest.

## Abbreviations

IAA: isolated iliac artery aneurysms
CIA: common iliac artery
IIA: internal iliac artery
CIIAA: isolated common and internal iliac artery aneurysms
CIAA: common iliac artery aneurysms
IIAA: internal iliac artery aneurysms
IBD: iliac branch device.
